# Transcriptomic immune profiling of ovarian cancers in paraneoplastic cerebellar degeneration associated with anti-Yo antibodies

**DOI:** 10.1038/s41416-018-0125-7

**Published:** 2018-06-14

**Authors:** Clément Vialatte de Pémille, Giulia Berzero, Mathilde Small, Dimitri Psimaras, Marine Giry, Maïlys Daniau, Marc Sanson, Jean-Yves Delattre, Jérôme Honnorat, Virginie Desestret, Agusti Alentorn

**Affiliations:** 1 Brain and Spine Institute (ICM), Experimental Neuro-Oncology Department, Inserm U 1127, CNRS UMR 7225, Sorbonne Universités, UPMC Univ Paris 06, 75013 Paris, France; 2 Neuroscience Consortium, University of Pavia, Monza Policlinico and Pavia Mondino, Pavia, Italy; 3 Institut NeuroMyogène, Equipe Synaptopathies et Autoanticorps (SynatAc), INSERM U1217/UMR CNRS, 5310 Lyon, France; 4 French Reference Center on Paraneoplastic Neurological Syndrome, Hospices civils de Lyon, Lyon, France; 5 University of Lyon, Université Claude Bernard Lyon 1, Lyon, France; 6 Department of Neurology 2, Division Mazarin, Hôpital Pitié Salpêtrière, AP-HP, 47 Boulevard Hôpital, 75013 Paris, France; 7 Brain and Spine Institute (ICM), iGenSeq, Hôpital Pitié Salpêtrière, 47 Boulevard Hôpital, 75013 Paris, France

**Keywords:** Microarrays, Gene regulatory networks

## Abstract

**Background:**

Paraneoplastic neurological syndromes are rare conditions where an autoimmune reaction against the nervous system appears in patients suffering from a tumour, but not linked to the spreading of the tumour. A break in the immune tolerance is thought to be the trigger.

**Methods:**

The transcriptomic profile of 12 ovarian tumours (OT) from patients suffering from paraneoplastic cerebellar degeneration (PCD) linked to anti-Yo antibodies (anti-Yo PCD OT) was compared with 733 ovarian tumours (OT control) from different public databases using linear model analysis.

**Results:**

A prominent significant transcriptomic over-representation of CD8+ and Treg cells was found in anti-Yo PCD OT, as compared to the OT control. However, the overall degree of immune cell infiltration was similar, according to the ESTIMATE immune score. We also found an under-representation of M2 macrophages in anti-Yo PCD OT. Furthermore, the differentially expressed genes were enriched for AIRE-related genes, a well-known transcription factor associated with a broad range of autoimmune diseases. Finally, we found that the differentially expressed genes were correlated to the transcriptomic profiling of the cerebellar structures.

**Conclusions:**

Our data pinpointed the enrichment of acquired immune response, particularly high density of CD8+ lymphocytes, and high-level expression of CDR-related antigens in anti-Yo PCD OT.

## Introduction

Paraneoplastic neurological syndromes (PNS) are rare conditions in which an autoimmune reaction against the nervous system appears in patients suffering from cancer, not linked to the spreading of the tumour. Paraneoplastic cerebellar degeneration (PCD) is one of the most common PNS. Its clinical hallmark is a subacute cerebellar ataxia, and is most frequently related to ovarian or breast cancer.^1^

Pathophysiology of PNS is incompletely solved, but some specific auto-antibodies recognising auto-antigens of the nervous system (e.g., anti-Yo antibodies) were found in the sera of patients, and are now included in the diagnostic criteria.^1^ Anti-Yo antibodies (Abs) recognise two Purkinje cells’ intracellular antigens, the 62 kD cerebellar degeneration related protein 2 (CDR2) and the 34 kD cerebellar degeneration related protein 1 (CDR1). In addition, other members of the CDR family, CDR2L share a high-sequence homology with CDR2, and anti-Yo antibodies can cross-react with both the antigens.^2^ Very recently, it has shown that CDR2L is overexpressed in the majority of anti-Yo PCD ovarian carcinomas and every PCD patient presented at least *CDR2L* gene-amplification/protein overexpression or *CDR2L* gene mutation, suggesting that each of these alterations might be sufficient to break the tolerance and trigger Yo disorders.^3^ While in vitro cytoxicity of anti-Yo antibodies has been suggested,^4^ reproduction of human disease in animal models have failed.^5^ On the other hand, several facts indicate an immune mediated disease: the presence of specific auto-antibodies and T cells recognising the same antigen,^6^ the immunisation of animal models with recombinant Yo antigen, even though no clinical nor histological abnormalities were found.^7^ To date, only few studies have directly explored the tumoural tissue, and it is generally accepted that PNS occurs at the fulcrum between the patient’s immune system and the tumour, where genetics and environment may play a role. On the patient scale, only human leucocyte antigen (HLA) have been explored, with negative results.^8^ However, very recently, a potential HLA genetic predisposition to anti-Yo PCD in the context of a specific cancer has been pinpointed.^9^

We hypothesise that antigen ectopic expression may contribute in the breakdown of immune tolerance. Therefore, transcriptomic analysis of anti-Yo PCD OT tissue may provide an insight of the pathways associated with this rare disease.

We propose here a comparative transcriptomic analysis of ovarian tumoural tissue from patients suffering from anti-Yo PCD OT, compared to a large series of different datasets of transcriptomic studies of ovarian cancer.

## Methods

### Recruitment and processing

To be included in the study, patients had to meet the following criteria: (i) presence of anti-Yo antibodies in the serum or the cerebrospinal fluid; (ii) histologically proven ovarian cancer with available tumour sample; (iii) PCD diagnosis according to the international criteria.^1^ All patients gave their informed consent. The French Reference Centre for PNS collected 12 formalin-fixed paraffin-embedded (FFPE) samples of tumours sampled prior to any systemic treatment. Samples were obtained from year 1998 to year 2015. Five sections were cut in each block and Qiagen® RNeasy FFPE kit was used to extract the RNA. The whole-tissue RNA was used for further analysis. Transcriptome analysis was performed via Affymetrix® HTA 2.0 microarray, which fits the best for FFPE samples,^10^ with a large probe per transcript coverage, reducing RNA degradation bias.

Data pre-processing, normalisation, differential expression and pathway analysis were made using the R language (version 3.2.3, 2015-12-10), using different packages from the Bioconductor.^11^ Details on the methods used in this study are provided in the Supplementary methods.

### Public data selection

Public data of ovarian tumours transcriptomes were obtained from The Cancer Genome Atlas (TCGA) OV dataset using level 3 data, ArrayExpress website and GEO-NCBI website.^12–14^ Because PCD affects less than 1 per 1000 ovarian cancer,^15^ we considered all the samples from the public data as non-PCD associated. Accordingly, transcriptomic data could classify our anti-Yo PCD OT samples correctly with an accuracy of 100% (95% confidence interval of 98–100%), supporting that transcriptomic data from the public dataset were composed of the OT controls. Further details on this analysis are provided in the Supplementary methods.

Furthermore, we also used 16 additional transcriptomic datasets with clinical data to analyse the potential clinical value of CDR2 and CDR2L expression on the survival of OT using curatedOvarianData Bioconductor package.^16^ Further details on the different public transcriptomic datasets are provided in the Supplementary data.

### Statistics

Normalisation and analysis methods are available as Supplementary data. We analysed transcriptomic data through different methods. First, we used CIBERSORT^17^ algorithm to determine and compare haematopoietic cells repartition within the samples (Fisher’s exact test). Second, we used ESTIMATE^18^ algorithm to assess tumour purity and to score the immune and stromal components, comparing it across samples (Wilcoxon’s test). Third, we used a single sample gene set enrichment analysis (ssGSEA) to establish the transcriptomic subtypes of each sample using the gene set described by Tothill.^19^ Fourth, in order to establish the consensus transcriptomic groups, we used consensusOV R package, according to four different transcriptomic classification algorithms (Supplementary Table 1).^20–23^ This approach improved the robustness of the proposed subtype classifiers, thereby providing reliable stratification of every sample of the clearly defined subtype. Fifth, we have performed meta-analysis using a fixed-effect model of several public transcriptomic OT studies using univariate coefficients of Cox model and multivariate Cox model, adjusting by debulking surgery, defined as residual tumour smaller than 1 cm and Federation of Gynecology and Obstetrics (FIGO) stage to establish the potential role of *CDR2* and *CDR2L* expression on the survival of OT using curatedOvarianData Bioconductor package.^16^ The results of the different studies are presented as forest plots (Supplementary Figure 1–3). Finally, we used data from the Allen brain atlas project^24^ to perform radio-genomic analysis, with family-wise error rate (FWER) set at 0.05.

## Results

### Description

Twelve samples from 12 female patients with anti-Yo Abs related-PCD aged 49–82 years (median 62) were used to perform the microarray (Table 1). Tumour diagnoses were high-grade serous carcinoma in ten patients, an ovarian high-grade clear-cell carcinoma (CCC) and a carcinosarcoma, according to the World Health Organisation (WHO) histologic classification of ovarian tumours. Tumour grading ranged from IA to IV, according to the International Federation of Gynecology and Obstetrics (FIGO) ovarian cancer staging. The most representative grade was III (50%, 6/12). Median delay between the symptoms onset and the diagnosis of the tumour was 2.6 months (range 1.4–72.4 months). Median time since collection was 11.6 years (range 1.4–18.6 years). RNA quality controls were satisfying and expected from FFPE tissues, with homogenous values (Supplementary Figure 4).

**Table 1:** Clinical data of the anti-Yo PCD cohort

Patient	Age (years)	Diagnosis	Grade	Delay between onset of symptoms and diagnosis of cancer (months)	Sample age (years)
1	54.8	HGSC	IV	1.4	5.1
2	70.5	HGSC	IA	1.6	7.2
3	54.7	HGSC	IV	-54.8	18.6
4	66.7	HGSC	IV	-16.3	12.4
5	50.2	HGSC	IIIC	5.1	14.1
6	72	HGSC	IV	2.5	12.2
7	62.8	HGSC	IIIC	2.6	13.5
8	80	HGSC	IIIC	-28.4	16.2
9	81.4	CCC	IA	-72.4	11.6
10	55.2	CS	IIIC	-3.5	4.4
11	62.3	HGSC	IIIC	1.6	9.6
12	51.2	HGSC	IIIA	6	1.4

*CCC* clear-cell carcinoma, *CS* carcinosarcoma, *HGSC* high-grade serous carcinoma

### Selection of datasets

Samples from TCGA,^25^ GSE51373,^26^ GSE69207,^27^ GSE66957 (unpublished data) and GSE63553 (unpublished data) were then included in the analysis (Supplementary Table 2 and Supplementary Figure 5). All samples were obtained before chemotherapy was administered. Because all datasets used for comparison were fresh frozen (FF) samples, we decided to remove the genes that were known to be differentially expressed within the paired samples from FFPE and FF origins.^28^ Our data confirmed these data, but also showed specific differential information unrelated to the samples’ origins, since 7.02% of first differentially expressed genes matched with the gene set in the literature (Supplementary Figure 6). After removal and normalisation, the final expression matrix had 9939 genes (Supplementary Table 3) and 745 samples.

### Differentially expressed (DE) genes

**Fig. 1:**
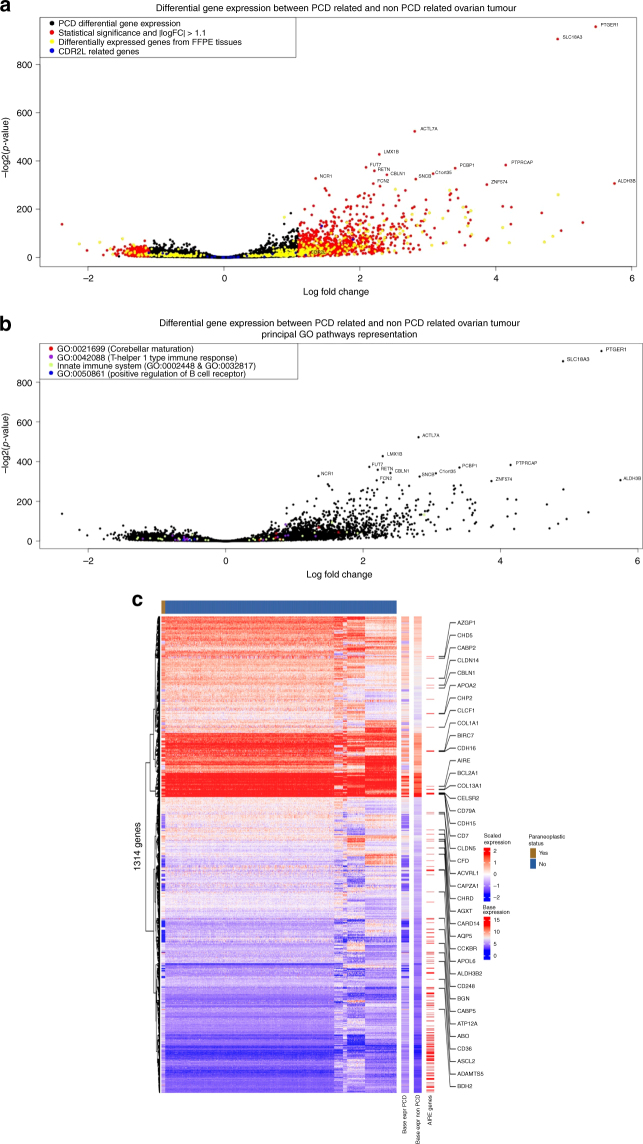
**a**, **b** Volcano plot of differentially expressed (DE) genes between paraneoplastic cerebellar degeneration (PCD) related ovarian tumours and control ovarian tumours. In **a**, coloured in red, genes with *p*-value over 0.05 and logfold change absolute value over 1.1, in yellow, genes know to be DE between fresh-frozen and formalin-fixed paraffin embedded samples and in blue, *CDR2L* gene and related genes. In **b**, the coloured plot are the genes included in following Gene Ontology terms: GO:0021699 (red), GO:0042088 (purple), GO:0002448, GO:0032817 (green), and GO: 0050861 (blue). Most significant gene names are plotted. **c** Heatmap of the 1314 differentially expressed genes (rows) and 12 ovarian cancer samples related with an anti-Yo paraneoplastic syndrome (top panel, indicated in brown) and 733 ovarian cancer samples from public databases (top panel in blue). The heatmap to the left indicates z-scales expression values, and two columns to the right (base expr PCD and base expr non-PCD) express the log2 base mean expression of the 1314 genes in the ovarian cancer samples related to an anti-Yo paraneoplastic syndrome and the rest of the samples, respectively. The third column on the right highlights the 171 AIRE-related genes within the differentially expressed gene set. For AIRE-related genes with relatively high expression levels (larger than 50% quantile of all genes), the gene name is indicated as text labels

A total of 5634 genes were differentially expressed between anti-Yo PCD OT samples and OT control samples: about 3093 and 2541 genes were up and downregulated, respectively (Fig. 1, Supplementary Table 4). Among them, 1314 differentially expressed genes had an absolute log fold change >1.1 and a *p*-value <0.001.

**Fig. 2:**
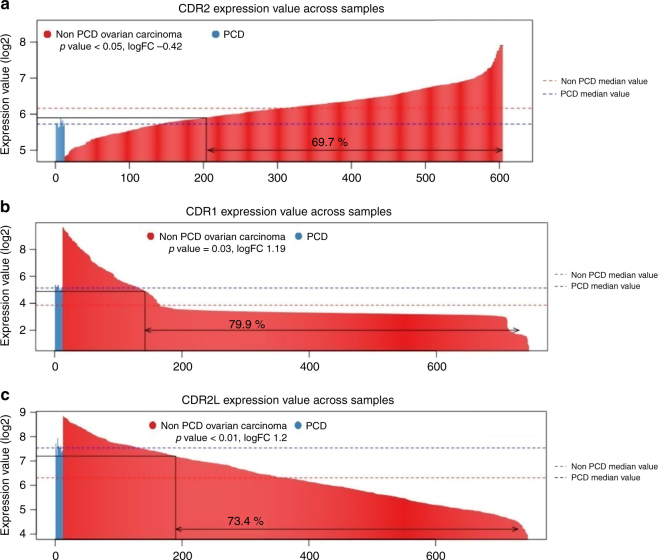
**a**, **c** Barplots representing log2 expression value of *CDR1* (**a**), *CDR2* (**b**), and *CDR2L* (**c**) genes across samples. Median values of the PCD samples and non-PCD samples are drawn. Proportions of the non-PCD samples exceeding the maximum PCD samples' expression value, or under minimum value avec written. Logfold change and *p* values are indicated under the legend

We looked for the genes related to the antigens of anti-Yo Abs: *CDR1*, *CDR2*, and *CDR2L*. *CDR2* gene was downregulated in anti-Yo PCD OT samples (logFC -0.42, *p*-value < 0.05) (Fig. 2a, Supplementary Table 4). *CDR1* and *CDR2L* genes were upregulated in anti-Yo PCD OT samples (Fig. 2b, c).

Three differentially expressed genes involved in regulatory T cells (Tregs) were differentially expressed: *FOXP3*, the Tregs promoter gene was upregulated (logFC 1.17, *p* < 0.001), and both *CTLA4* and *ICOS* genes, from the CD28 family, responsible for Tregs suppressive functions were downregulated (logFC −0.57 and −0.89, respectively, *p* < 0.05).

The autoimmune regulator (AIRE), a gene involved in negative thymic T lymphocytes selection, was upregulated in anti-Yo PCD OT samples (logFC 1.62, *p* < 0.001). Because *AIRE* is the master regulator of autoimmune responses, we further analysed the differentially expressed genes that were transcriptionally regulated by *AIRE*, using a previously described gene list of the *AIRE*-related genes.^29^ Surprisingly, among the 1314 differentially expressed genes, 171 (13%) were enriched with *AIRE*-related genes (hypergeometric test, FDR *p*-value < 0.001) (Fig. 1c, Supplementary methods).

**Fig. 3:**
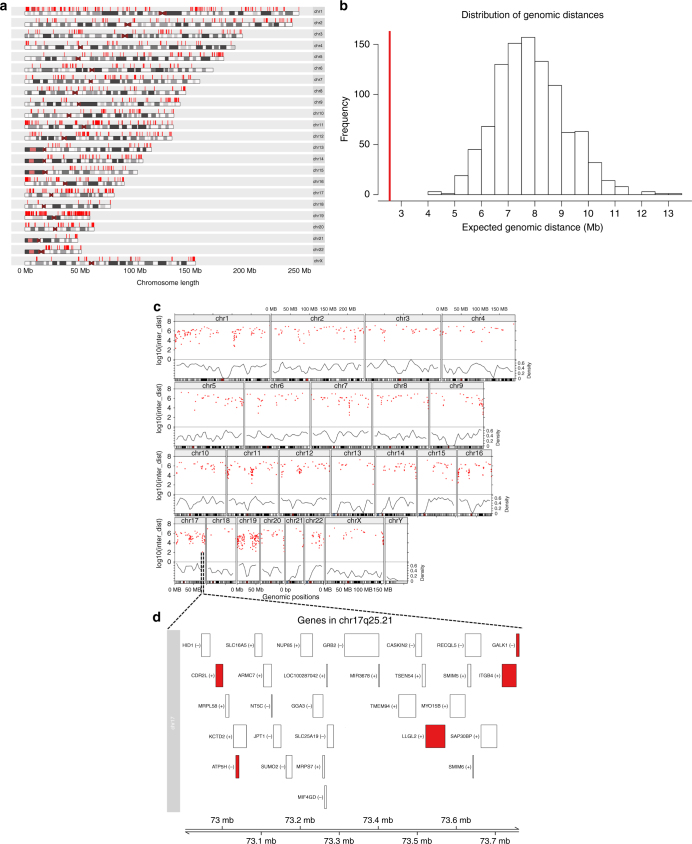
1314 differently expressed genes when comparing ovarian samples associated with an anti-Yo PNS vs. the public transcriptomic databases of high-grade ovarian cancer. **a** karyogram depicting genomic localisation of the differentially expressed genes. **b** Distribution of the expected median genomic distance between the two genes in the genome (based on 1000 permutations selecting random sets of genes of the same size as in the list of the differentially expressed gene set). The red line depicts the median distance observed for 1314 genes belonging to the differentially expressed genes, which deviates from the null model (FDR = 5%). **c** Rainfall plot showing the gene-gene distance density (red dots), in accordance to the expected gene density (black line). **d** Zoom area of the highest density of differentially expressed genes next to *CDR2L* gene in the chr17q25.21 cytoband

We calculated the median genomic distance between each gene to its nearest gene neighbour with the differentially expressed (DE) gene list. The DE gene list was located in significant genomic proximity (FDR of 5%) (Fig. 3), suggesting a non-random spatial distribution of the DE genes. Further details on the methods used are provided as Supplementary data. Despite being disperse across the genome, numerous genes were densely clustered in specific loci (Fig. 3c). We visualised the gene–gene inter-distance (Supplementary data) using a rainfall plot in order to identify a hotspot of clustered co-expressed DE genes (Fig. 3b). Importantly, the region with the highest density of DE genes was close to *CDR2L* locus in the cytoband 17q25.21 (Fig. 3d).

### Pathway analysis

**Fig. 4:**
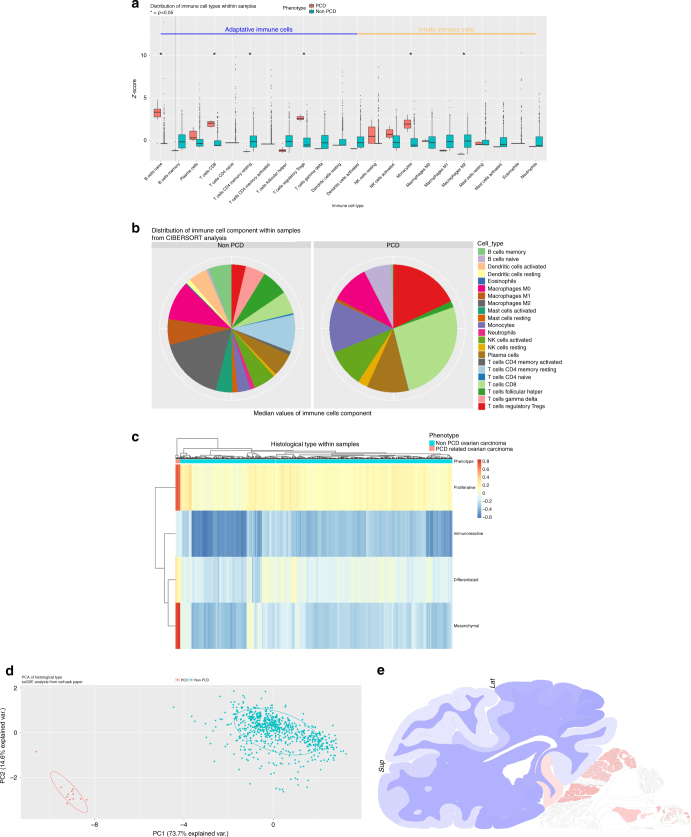
**a** Boxplots with *z*-score of the distribution of the different cell types between PCD and non-PCD related ovarian tumour samples. Analysis was performed with CIBERSORT algorithm. Classification regarding adaptive immune system (on the left) and innate immune system (on the right) was made. Statistical significance is shown by “*”, and significant cells are written in bold. **b** pie chart representing the distribution of the immune cell types (mean values) in non-PCD samples (on the left) and PCD samples (on the right). Data from CIBERSORT analysis. **c** Heatmap of the histological subtypes scores. Annotation on columns correspond to the samples' phenotypes (PCD or non-PCD samples). Data from single sample gene set enrichment analysis on gene sets from the literature(20). Hierarchical clustering was used to cluster the samples. **d** Principal component analysis (PCA) was the histological subtypes results matrix. Plot using first and second PCA (explaining respectively 73.7 and 14.6% of variance) and coloured membership of PCD samples (blue) and non-PCD samples (orange). **e**: heatmap of *CDR1*, *CDR2* and *CDR2L* genes expression across the brain. Data from the brainscope website using Allen brain institute project data. Brain is shown from coronal view through the cerebellum, with a 90° left rotation. Colour legend indicates expression value going from blue to red scale

Pathway analysis showed 1751 statistically significant Gene-Ontology and KEGG terms, mostly part of biological process, among which 132 terms were of biological interest regarding both the immune system and the CNS (Fig. 4, Supplementary Figures 7, Supplementary Table 5). The upregulated pathways were mostly a part of the innate immune system (*p* = 0.01, Odds ratio 6.2 [95% CI, 1.6–26.8]). Forty terms were related to the CNS, and 92.5% of them were upregulated. Interestingly, within the CNS enriched pathways, three were related to hindbrain maturation, among which the cerebellar cortex maturation (GO:0021699), with genes *ARCN1*, *CEND1* and *RERE*. To further characterise the brain transcriptomic representation of the DE gene list, we used ABAEnrichement package,^30^ to perform gene set expression enrichment analysis in the adult human brain using the Allen Brain Atlas dataset. Giving our list of DE genes, weighted by their logFC, the results showed five statistically (family-wise error rate, FWER <0.05) represented brain structures, all located within the cerebellum (Supplementary Table 6). In addition, *CDR1*, *CDR2* and *CDR2L* were mostly expressed in the cerebellum (Fig. 4e).

### Immune cell infiltration analysis using transcriptomic data

We applied a CIBERSORT *p*-value threshold of 0.1 to integrate the samples in comparative analysis, according to the small size of our samples. Naïve B cells (6.31% vs. 0.3%, *p* < 0.05), T CD8 cells (25.8% vs. 4.1%, *p* < 0.001), Tregs (16.32% vs. 1.8%, *p* < 0.001) and monocytes (11.68% vs. 1.86%, *p* < 0.05) were overrepresented in the anti-Yo PCD OT samples, whereas T CD4 memory resting cells (0.03% vs. 8.07%, *p* < 0.05) and M2 macrophages (0.78% vs. 18.99%, *p* < 0.001) were underrepresented (Fig. 4a, b, Supplementary Table 7). The ESTIMATE analysis showed no statistical differences among the samples, according to the stroma, immune and ESTIMATE scores, as well as the purity score (Supplementary Table 8).

Analysis of the histological subtype through transcriptomic data, using ssGSEA, showed that all PCD samples were of mesenchymal subtype, whereas the non-PCD samples were more often proliferative (*p* < 0.001) (Fig. 4c). Principal component analysis (PCA) was able to correctly segregate the samples' origins from the histological scores, with 73.7% of variance for the first component analysis (Fig. 4d).

### The potential impact on survival of *CDR2* and *CDR2L* expression

In order to establish the potential role of *CDR2* and *CDR2L* expression on overall survival of OT patients, we have performed a meta-analysis using a large list of public transcriptomic dataset (Supplementary Table 9). Interestingly, *CDR2* expression was associated with a worse prognosis in Cox univariate (Hazard ratio (HR) = 1.07 [95% confidence interval, CI, 1.01–1.13], *p*-value = 0.01 and multivariate analysis (HR = 1.07 [1–1.15]), *p*-value = 0.045 (Supplementary Figure 1 and Supplementary Fig. 2, respectively). Conversely, the expression of *CDR2L* did not have independent prognostic value (Supplementary Figure 3).

## Discussion

We have performed a transcriptomic analysis of 12 anti-Yo PCD OT, compared to a large transcriptomic series of 733 OT control samples from different public databases. The transcriptomic profile of anti-Yo PCD OT was remarkably different. We have identified a prominent transcriptomic overrepresentation of CD8+ and Treg, and also a significant underrepresentation of M2 macrophages in ovarian cancer samples related with an anti-Yo PNS. Interestingly, the DE genes were enriched for AIRE-related genes. Finally, we also found a non-random distribution of DE genes genome-wide, with the highest density of DE genes next to *CDR2L* in the chr17q25.21 cytoband.

Previously, it has been described that an overexpression of Human epidermal growth factor receptor 2 (HER2) at protein level in breast tumours is associated with anti-Yo PCD.^31^ We did not find a clear overexpression of HER2 at gene level in anti-Yo PCD OT samples. Accordingly, a recent study also analysing anti-Yo PCD OT samples did not find HER2 overexpression in anti-Yo PCD OT samples at protein level.^3^ These results suggest that the oncogenesis mechanisms related to OT or breast cancer with anti-Yo PCD are different.

Immune system involvement is largely present in PCD pathophysiology through multiple arguments, and one step of the immune tolerance break is considered to be the ectopic expression of a CNS antigen by the tumoural cells. Although it must be a great part of the PNS trigger, it cannot explain the whole pathophysiology since CDR2 and CDR2L antigens are found widely in ovarian tumours.^32,33^ The fact that *CDR2* is mostly regulated at the post-transcriptional level may explain why we failed to show a differential expression at RNA.^34^ On the other hand, *CDR1*, the other antigen recognised by the anti-Yo antibodies, and *CDR2L*, are upregulated in anti-Yo PCD OT samples (logFC 1.19 and 1.2 respectively, adjusted *p* < 0.05). We also showed that the *AIRE* gene, responsible for negative selection of T cells and implicated in a broad range of autoimmune diseases was upregulated in anti-Yo PCD OT (logFC 1.62, *p* < 0.001). Our data also showed that several CNS antigens were presumably overexpressed by PNS tumours, among which specific cerebellar antigens coded by the genes *RERE*, *CBLN1*, and *CCDC88C*. These results were consistent with the Allen Brain Atlas expression data, with differentially expressed genes being statistically correlated with cerebellar structures (Supplementary Table 6). Therefore, it is tempting to speculate that “transcriptomic mimicry” between these brain structures and anti-Yo PCD OT samples may be also associated with the involvement of the cerebellum degeneration in anti-Yo paraneoplastic syndrome.

The ESTIMATE analysis suggests that there is no difference in the degree of immune infiltration. However, we found important differences in both the cell type and abundance of immunological cells using CIBERSORT analysis. This could suggest a shift in the immune response that may justify that the immune cells involved in the acquired immunity were more represented in anti-Yo PCD OT samples such as Tregs, naive B cells and T CD8+ cells. Furthermore, CD8+cells infiltration was also found using immunohistochemistry and FACS analysis in a submitted study of anti-Yo PCD OT compared to OT controls.^3^ Intratumoural CD8+ infiltration samples has been associated with good prognosis in high-grade serous ovarian cancer.^35–37^ Besides, tertiary lymphoid structures, tumour-associated lymph node-like with a structure similar to secondary lymphoid organs, have been described in ovarian cancer with high density of T-lymphocytes and B-lymphocytes infiltration and their presence has been associated with the generation of tumour-specific T cells.^38,39^ Remarkably, the concentration of autoantibodies has been correlated to the production of Tregs and with the presence of other paraneoplastic syndromes.^40,41^

It has been recently shown a cross talk between *AIRE* regulated gene expression and Treg cells. Strikingly, *AIRE*-dependent thymic development of tumour-associated Tregs was found in a mouse model of prostate cancer.^42^ More recently, it has also been shown that *AIRE* enforces immune tolerance by directing auto-reactive T cells into the Treg cell lineage.^43^ Moreover, intratumoural *AIRE* expression has been associated with acute inflammation in other epithelial tumours.^44^ In addition, it has been described that extrathymic *AIRE*-expressing cells are also present in secondary lymphoid organs and may be involved in immune tolerance.^45^ These data could suggest that the *AIRE* related immune response might be associated with the autoimmune response associated to anti-Yo Abs but also with the tumour-infiltrating lymphocytes profiling.

We also found a non-random distribution of the DE gene list (Fig. 3a). It is noteworthy to mention that transcriptomic “hot spots” (i.e., regions with a high density of differentially expressed genes) have been also described in other autoimmune disease in loci with pathogenetic association.^46^ One possible mechanism for this clustered expression could be local chromatin configuration that would allow the ectopic expression of neighbouring genes, irrespective of their regulation in peripheral tissues.^47^ Chromatin re-modelling can affect nearby genes on the same chromosome but also genes nearby in three-dimensional architecture of the nucleus. A correlation between gene expression and co-localisation in transcription factors has been described for lineage-specific gene regulation.^48^ In order to directly assess the chromatin state for differentially expressed genes, ATAC-Seq studies are warranted, which is based on the preference of the TN5 transposase to integrate into un-compacted chromatin and thus allows a direct measurement of chromatin accessibility.^49^

Previous studies have shown contradictory results with regard of CDR2 expression in PCD patients.^33,50^ In addition, the role of the Yo-antibody in the pathogenesis of PCD is controversial but some evidence suggest that CDR antibody internalisation causes dysregulation of cell calcium homoeostasis and also Purkinje cell death and this phenomenon was not simply due to intraneuronal antibody accumulation.^51,52^ In addition, the events that trigger anti-Yo antibody response are not well elucidated. However, a recent study has identified that all patients with anti-Yo PCD OT harbour mutations and/or gains in *CDR2* and/or *CDR2L* genes, which could lead to immune tolerance breakdown and autoimmunity.^3^

Our study has limitations worth noting: we firstly used FFPE samples due to the extreme rarity of PNS; however, we avoided batch effect and comparison bias using powerful normalisation methods, and by removing known genes to be differentially expressed between FFPE and FF paired samples.^28^ Using multiple sources of transcriptomic data for ovarian cancers as a merged control also helped to reduce this bias. Finally, this represents a small cohort with only 12 anti-Yo PCD OT, but this is the first transcriptomic study in this rare entity. Furthermore, another independent recent study has confirmed our major findings using immunohistochemistry and fluorescence-activated cell sorting (FACS) analysis.^3^

In conclusion, our study improves our current knowledge of the biological pathways involved in PNS using high-throughput transcriptome data. Our study pinpoints the involvement of a broad range of different immune cells in PNS. We hypothesise that antigen ectopic expression may contribute in the break of the immune tolerance, with a larger representation than the antigen recognised by the known antibody. Further studies, focused on what emerged of our study will be needed to deepen the comprehension of the exact mechanisms of PNS disorders.

## Electronic supplementary material

Supp Methods

Supp Figure 1

Supp Figure 2

Supp Figure 3

Supp Figure 4

Supp Figure 5

Supp Figure 6

Supp Figure 7

Supp Table1

Supp Table2

Supp table3

Supp Table4

Supp Table5

Supp Table6

Supp Table7

Supp Table8

Supp Table9

## Availability of data and material

Raw expression data have been deposited to ArrayExpress database (www.ebi.ac.uk/arrayexpress) under accession number E-MTAB-5942. The R scripts of the most important steps of these analyses, and some of the supplementary data are available online with the following link (https://figshare.com/projects/CVDP_antiYo_PCD_transcriptome/23503).
