# Supplementary material for: Transcriptomic immune profiling of ovarian cancers in paraneoplastic cerebellar degeneration associated with anti-Yo antibodies
Source: Br J Cancer. 2018 Jun 14;119(1):105–13. doi: 10.1038/s41416-018-0125-7 (PMC6035206; doi:10.1038/s41416-018-0125-7)
Supplement: Supplementary file 1 — Supp Methods [file 41416_2018_125_MOESM1_ESM.doc]

**Comparative transcriptomic analysis of ovarian cancers in paraneoplastic cerebellar degeneration associated with anti-Yo antibodies**

*Clément Vialatte de Pémille^1^, Giulia Berzero^1^, Dimitri Psimaras^1-2^, Marine Giry^1^, Maïlys Daniau^1,3^, Marc Sanson^1-2^, Jean-Yves Delattre^1-2^, Jérôme Honnorat^4-6^, Virginie Desestret^4-6^, Agusti Alentorn^1-2^*

1 - Brain and spine institute (ICM), experimental neuro-oncology department, Hôpital Pitié Salpêtrière, 47 Boulevard Hôpital, 75013 Paris, France.

2 - Department of Neurology 2, division Mazarin, Hôpital Pitié Salpêtrière, AP-HP, 47 Boulevard Hôpital, 75013 Paris, France.

3 - Brain and spine institute (ICM), iGenSeq, Hôpital Pitié Salpêtrière, 47 Boulevard Hôpital, 75013 Paris, France.

4 - Institut NeuroMyogène, Equipe Synaptopathies et Autoanticorps (SynatAc), INSERM U1217/UMR CRS 5310, Lyon, France

5 - French Reference Center on Paraneoplastic Neurological Syndrome, Hospices civils de Lyon, Lyon, France

6 - University of Lyon, Université Claude Bernard Lyon 1, Lyon, France

Corresponding author: Agusti Alentorn, Department of Neurology 2brain and spine institute (ICM), experimental neuro-oncology department, Hôpital Pitié Salpêtrière, 47 Boulevard Hôpital, 75013 Paris, France. Email : agusti.alentorn@gmail.com or [agusti.alentorn@aphp.fr](mailto:agusti.alentorn@aphp.fr)

**Supplementary methods**

*Packages and versions:*

Loading of raw files was made using pd.hta.2.0 package (version 3.12.1). Background subtraction, quantile normalization and summarization using a log2 expression scale were made with oligo package^1^ (version 1.34.2), using Robust Multi-Array Average (RMA) method. Normalization was made on 70 553 probes. A linear model was used to assess the quality of the transcriptomic data using the fitProbeLevelModel function. Annotation was made with packages AnnotationDBI^2^ (version 1.38.1) and org.hs.eg.db (version 3.2.3).

*Normalization*:

To avoid batch effect, normalization between datasets was performed using a cyclic loess nonlinear method with a fast implementation^3^. Comparability across datasets was assessed using several methods among which hierarchical clustering, principal component analysis (PCA), Pearson’s correlation analysis, before and after normalization.

*Machine learning classification of transcriptomic profiles:*

We used the classification and regression training (caret package^4^, version 6.0-77). We used the entire list of common transcriptome dataset and we splitted into 60% sample of training and the rest as a test cohort considering the variable PCD to balance the distribution of class distributions between the splits. We then used glmnet package^5^ (version 2.0-10) that fits a generalized linear model via penalized maximum likelihood. We used a grid of values for the regularization parameter that maximizes the results. We also performed a second tuning parameter, the mixing percentage, that represents the elastic-net penalty. We used the trControl function to choose the optimal values of the tuning parameters using a 10-fold cross-validation. Finally, we predicted in the test cohort the identified optimal model using the predict method. Accuracy (true positives + true negatives) / (true positives + false positives + false negatives + true negatives) was obtained using the confusionMatrix function.

*Statistical analysis:*

*Differential gene expression and pathway analysis:*

Differential gene expression analysis was done by using a linear model implemented in the limma^6^ package (version 3.26.9). Benjamini-Hochberg^7^ (BH) method was used to correct multiple testing. Pathway analysis were done on the following Bioconductor packages: GSVA^8^ (version 1.18.0), Gage^9^ (version 2.26.1), ClusterProfiler^10^ (version 2.4.3). We also used the online software Enrichr^11^ and Gorilla^10^. False discovery rate (FDR) and p-value were respectively set to 0.05 when logFC was included in the analysis and 0.1 when unweighted gene names were used. GO (Gene Consortium Ontology) and KEGG (Kyoto Encyclopaedia for Genes and Genomes) ontologies were used in pathway analysis. Go terms summary and correlation analysis were made using G-SESAME^13^ online software, using Pearson correlation method. We Protein-protein network analysis was conducted on the online software NetworkAnalyst^14^ and the R package dnet^15^ (version 1.0.10). We used JASPAR^16^ database to analyze interaction of *CDR2L* and its related genes with transcription factors. We used BioGrid^17^ (version 3.4) and String^18^ (version 10.5) software to assess *CDR2L* related genes and proteins.

*AIRE related gene analysis:*

Hypergeometric testing was performed on all common genes between studies to assess whether the observed overlap was significantly greater than expected by chance alone. In the context of this test, we defined “enrichment ratio” as : enrichment ratio = observed gene counts / expected gene counts. To assess significance of overlap amongst differentially expressed gene list and AIRE related genes, we performed a bootstrap to estimate the p-value. For this, we used a total of two variables, representing differentially expressed gene list and AIRE related genes. Each variable constituted a vector of positions of length n matching the number of significant genes (n = 1314, for the DE gene list, n = 171, for the AIRE related gene list). We randomly allocated the positions in each variable vector to a vector of positions that represented the total number of genes in our analysis (n = 9035). We then counted the positions (i.e. rows) that were true for all two variables and repeated this process 106 times. We used the distribution thus generated to evaluate the fraction of permuted counts that was greater than our observed overlap and used this to approximate the p-value.

*Analysis of non-random spatial distribution of DE genes*:

We constructed a null model that allowed us to estimate the expected median genomic distance between genes given the size of this gene list. In order to visualize these effects, we plotted the localization of the differentially expressed gene list in a karyogram representation *(Figure 4A).* We also calculated the density of distribution of genomic regions that contained the differentially expressed gene list. The density for a region was defined as the percentage of a genomic window that was covered by the input genomic windows. We then calculated the gene-gene inter-distance (i.e. for a region there was a distance to the previous region and also there was a distance to the next region).

**Bibliography:**

1. Carvalho BS, Irizarry RA. A framework for oligonucleotide microarray preprocessing. *Bioinforma Oxf Engl*. 2010;26(19):2363-2367. doi:10.1093/bioinformatics/btq431.

2. AnnotationDbi. Bioconductor. http://bioconductor.org/packages/AnnotationDbi/. Accessed July 20, 2017.

3. Ballman KV, Grill DE, Oberg AL, Therneau TM. Faster cyclic loess: Normalizing RNA arrays via linear models. *Bioinformatics*. 2004;20(16):2778-2786. doi:10.1093/bioinformatics/bth327.

4. Building Predictive Models in R Using the caret Package | Kuhn | Journal of Statistical Software. doi:10.18637/jss.v028.i05.

5. Regularization Paths for Generalized Linear Models via Coordinate Descent | Friedman | Journal of Statistical Software. doi:10.18637/jss.v033.i01.

6. Ritchie ME, Phipson B, Wu D, et al. limma powers differential expression analyses for RNA-sequencing and microarray studies. *Nucleic Acids Res*. 2015;43(7):e47. doi:10.1093/nar/gkv007.

7. Hochberg Y, Benjamini Y. More powerful procedures for multiple significance testing. *Stat Med*. 1990;9(7):811-818.

8. Hänzelmann S, Castelo R, Guinney J. GSVA: gene set variation analysis for microarray and RNA-seq data. *BMC Bioinformatics*. 2013;14:7. doi:10.1186/1471-2105-14-7.

9. Luo W, Friedman MS, Shedden K, Hankenson KD, Woolf PJ. GAGE: generally applicable gene set enrichment for pathway analysis. *BMC Bioinformatics*. 2009;10:161. doi:10.1186/1471-2105-10-161.

10. Yu G, Wang L-G, Han Y, He Q-Y. clusterProfiler: an R package for comparing biological themes among gene clusters. *Omics J Integr Biol*. 2012;16(5):284-287. doi:10.1089/omi.2011.0118.

11. Chen EY, Tan CM, Kou Y, et al. Enrichr: interactive and collaborative HTML5 gene list enrichment analysis tool. *BMC Bioinformatics*. 2013;14:128. doi:10.1186/1471-2105-14-128.

12. Eden E, Navon R, Steinfeld I, Lipson D, Yakhini Z. GOrilla: a tool for discovery and visualization of enriched GO terms in ranked gene lists. *BMC Bioinformatics*. 2009;10:48. doi:10.1186/1471-2105-10-48.

13. Du Z, Li L, Chen C-F, Yu PS, Wang JZ. G-SESAME: web tools for GO-term-based gene similarity analysis and knowledge discovery. *Nucleic Acids Res*. 2009;37(Web Server issue):W345-349. doi:10.1093/nar/gkp463.

14. Xia J, Gill EE, Hancock REW. NetworkAnalyst for statistical, visual and network-based meta-analysis of gene expression data. *Nat Protoc*. 2015;10(6):823-844. doi:10.1038/nprot.2015.052.

15. Fang H, Gough J. The “dnet” approach promotes emerging research on cancer patient survival. *Genome Med*. 2014;6(8):64. doi:10.1186/s13073-014-0064-8.

16. Sandelin A, Alkema W, Engström P, Wasserman WW, Lenhard B. JASPAR: an open-access database for eukaryotic transcription factor binding profiles. *Nucleic Acids Res*. 2004;32(Database issue):D91-94. doi:10.1093/nar/gkh012.

17. Chatr-Aryamontri A, Oughtred R, Boucher L, et al. The BioGRID interaction database: 2017 update. *Nucleic Acids Res*. 2017;45(D1):D369-D379. doi:10.1093/nar/gkw1102.

18. Szklarczyk D, Morris JH, Cook H, et al. The STRING database in 2017: quality-controlled protein-protein association networks, made broadly accessible. *Nucleic Acids Res*. 2017;45(D1):D362-D368. doi:10.1093/nar/gkw937.
