# Supplementary figures and images for: Transcriptomic immune profiling of ovarian cancers in paraneoplastic cerebellar degeneration associated with anti-Yo antibodies
Source: Br J Cancer. 2018 Jun 14;119(1):105–13. doi: 10.1038/s41416-018-0125-7 (PMC6035206; doi:10.1038/s41416-018-0125-7)

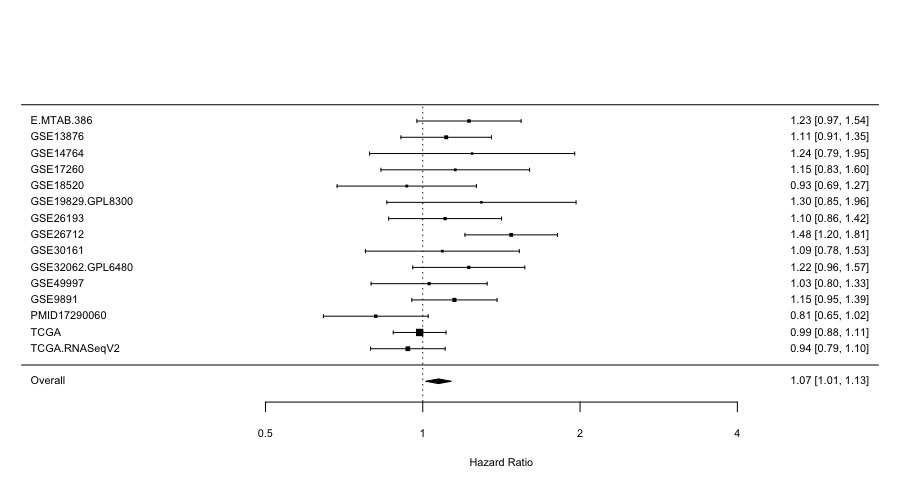

Supplement: Supplementary file 2 — Supp Figure 1 [file 41416_2018_125_MOESM2_ESM.tif]

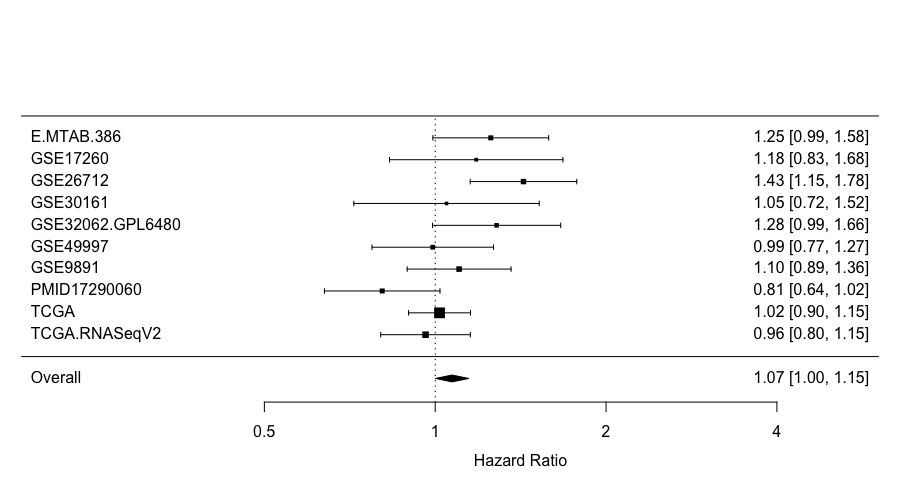

Supplement: Supplementary file 3 — Supp Figure 2 [file 41416_2018_125_MOESM3_ESM.tif]

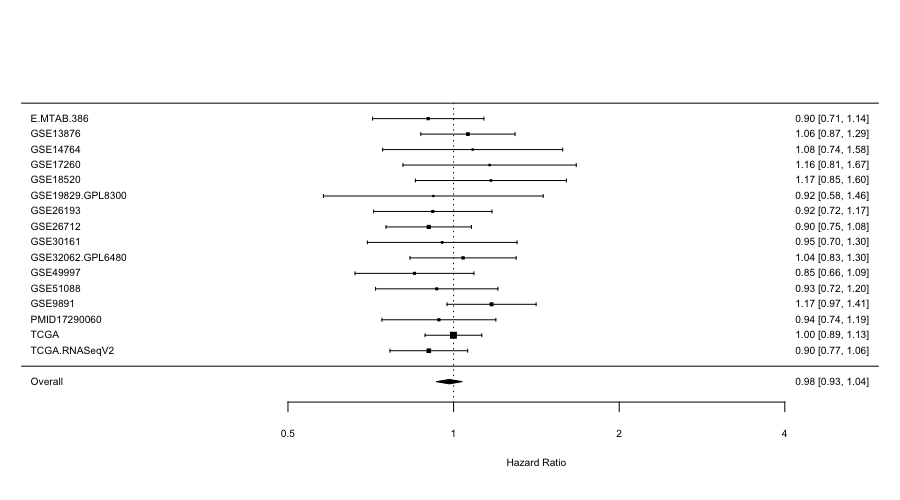

Supplement: Supplementary file 4 — Supp Figure 3 [file 41416_2018_125_MOESM4_ESM.tif]

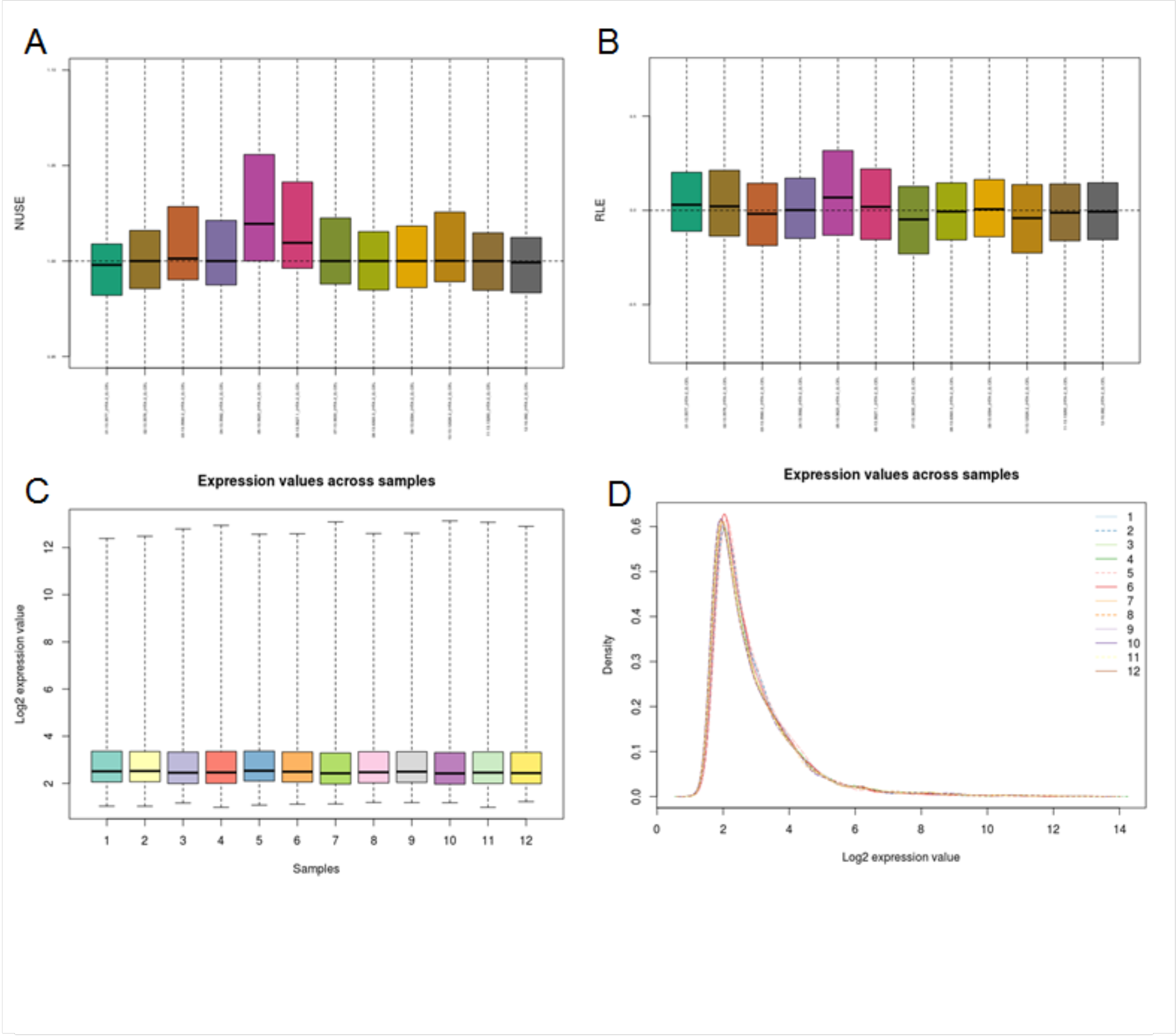

Supplement: Supplementary file 5 — Supp Figure 4 [file 41416_2018_125_MOESM5_ESM.tif]

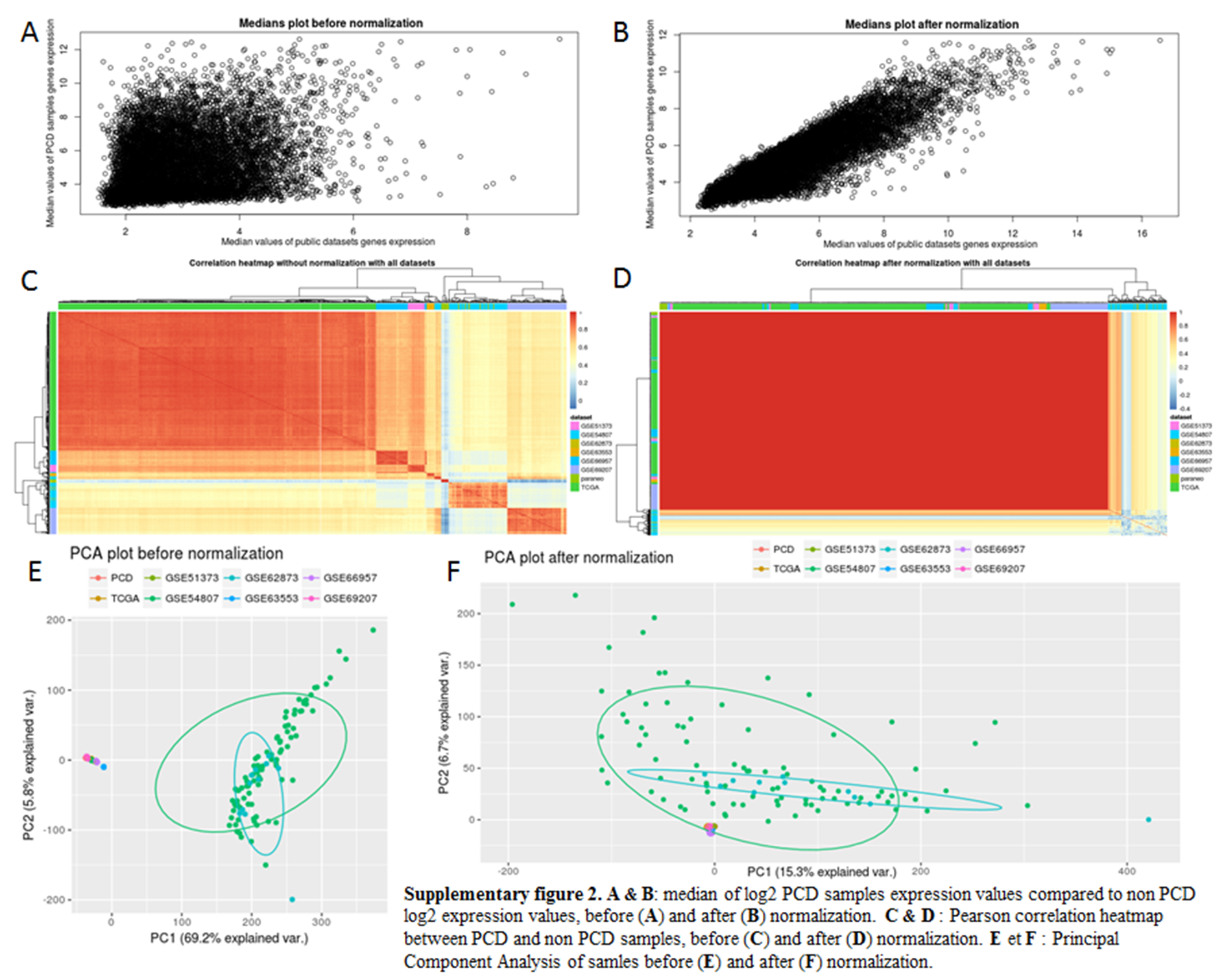

Supplement: Supplementary file 6 — Supp Figure 5 [file 41416_2018_125_MOESM6_ESM.tif]

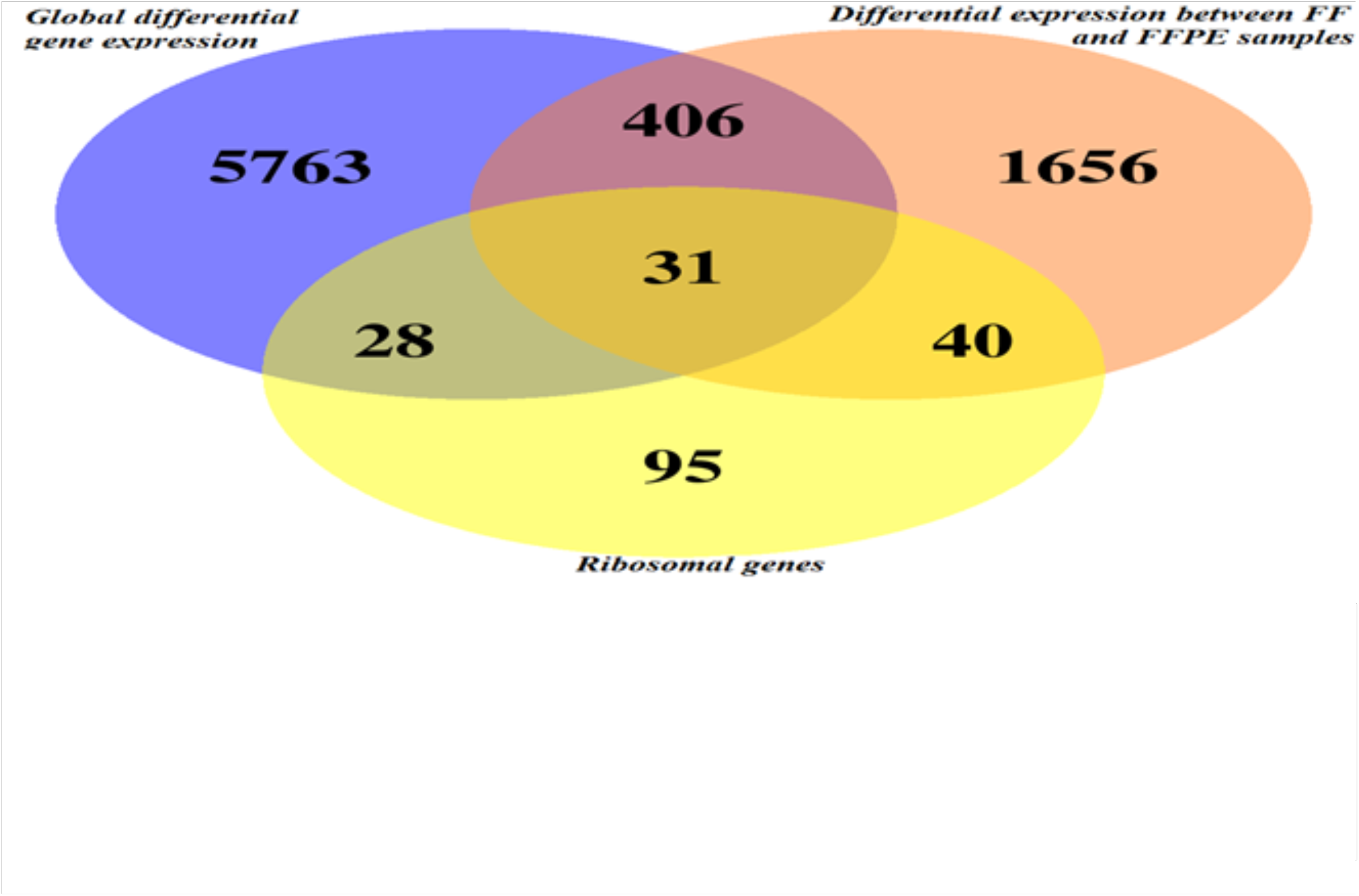

Supplement: Supplementary file 7 — Supp Figure 6 [file 41416_2018_125_MOESM7_ESM.tif]

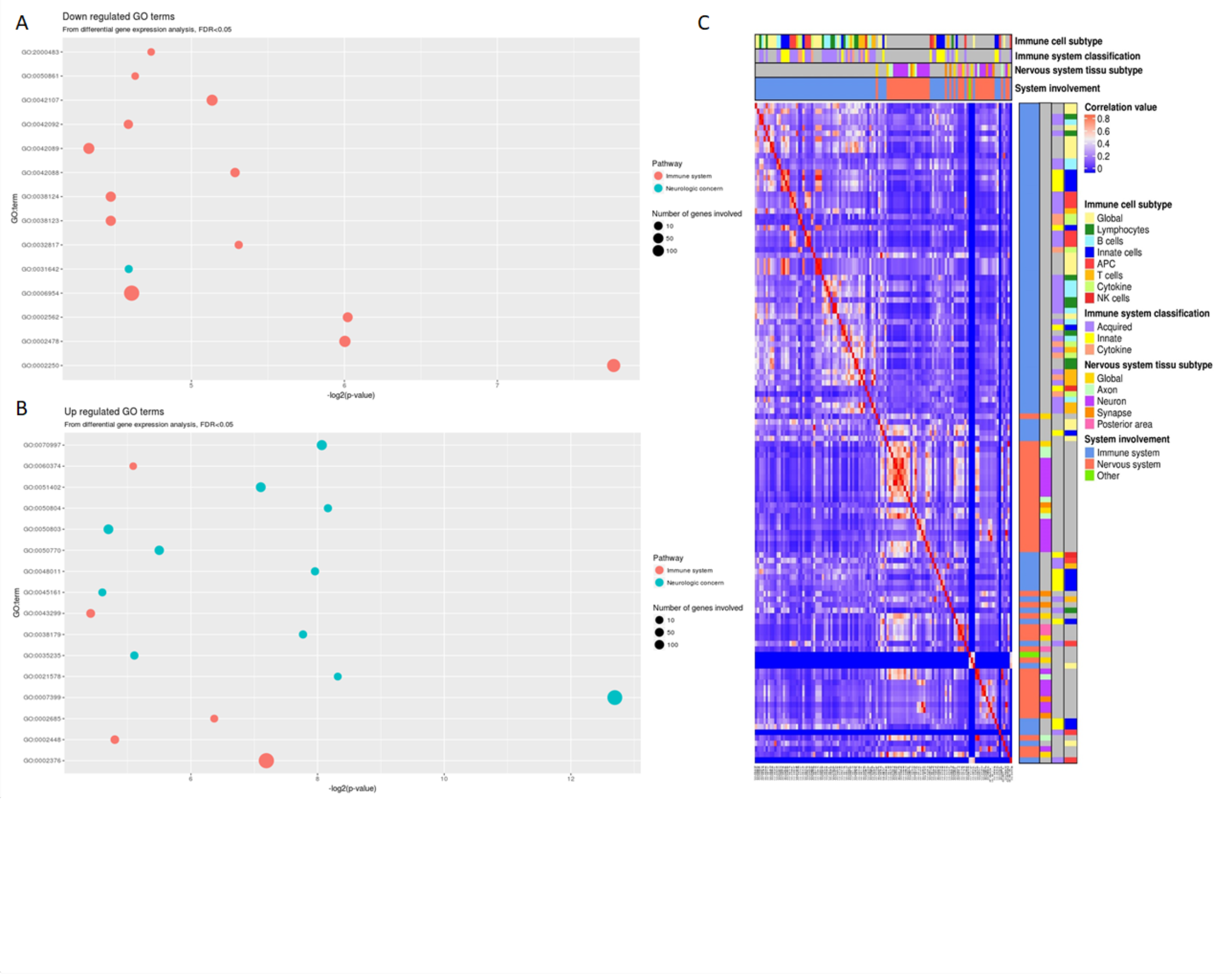

Supplement: Supplementary file 8 — Supp Figure 7 [file 41416_2018_125_MOESM8_ESM.tif]
