# Supplementary material for: Transcriptomic immune profiling of ovarian cancers in paraneoplastic cerebellar degeneration associated with anti-Yo antibodies
Source: Br J Cancer. 2018 Jun 14;119(1):105–13. doi: 10.1038/s41416-018-0125-7 (PMC6035206; doi:10.1038/s41416-018-0125-7)
Supplement: Supplementary file 10 — Supp Table2 [file 41416_2018_125_MOESM10_ESM.doc]

| **Reference** | **Plateform** | **Number of samples** | **Conservation method** | **Analysed** |
| --- | --- | --- | --- | --- |
| **GSE51373** | Affymetrix° U133 Plus 2.0 | 28 | FF | Y |
| **GSE54807** | Affymetrix° Exon 1.0 ST | 11 | FFPE | N |
| **GSE69207** | Affymetrix° Exon 1.0 ST | 100 | FF | Y |
| **TCGA** | Affymetrix° U133 Plus 2.0 | 535 | FF | Y |
| **GSE62873** | Affymetrix° U133 Plus 2.0 | 99 | FF | N |
| **GSE66957** | Affymetrix° U133 Plus 2.0 | 57 | FF | Y |
| **GSE63553** | Affymetrix° Exon 1.0 ST | 13 | Cell line | N |

FF: fresh-frozen, FFPE: formalin fixed paraffin embedded, TCGA: the cancer genome atlas
